# Evaluation of antibody drug delivery efficiency via nebulizer in various airway models and breathing patterns

**DOI:** 10.1186/s40360-023-00711-9

**Published:** 2023-12-01

**Authors:** Soon Woo Hong, Kyung Hwa Chang, Chang Jae Woo, Ho Chul Kim, Bong Seop Kwak, Bong Joo Park, Ki Chang Nam

**Affiliations:** 1 Department of Medical Engineering, Dongguk University College of Medicine, Goyang-si, 10326 Gyeonggi-do Korea; 2 Department of Radiological Science, Eulji University, Seongnam-si, 13135 Gyeonggi-do Korea; 3 Department of Electrical & Biological Physics and Institute of Biomaterials, Kwangwoon University, Seoul, 01897 Korea; 4 Office of Technology Transfer, National Cancer Center, Goyang-si, 10408 Gyeonggi-do Korea

**Keywords:** Nebulizer, IgG, Drug delivery, Respiratory disease, Breathing simulator

## Abstract

**Background:**

Nebulizers are commonly used to treat respiratory diseases, which are a major cause of morbidity and mortality. While inhalation therapy with antibodies has been evaluated in preclinical studies and clinical trials for respiratory diseases, it has not yet been approved for treatment. Moreover, there is limited information regarding the delivery efficiency of therapeutic antibodies via nebulizer.

**Methods:**

In this study, the nebulization characteristics and drug delivery efficiencies were compared when immunoglobulin G (IgG) was delivered by five nebulizers using two airway models and five breathing patterns. The study confirmed that the delivered dose and drug delivery efficiency were reduced in the child model compared to those in the adult model and in the asthma pattern compared to those in the normal breathing pattern.

**Results:**

The NE-SM1 NEPLUS vibrating mesh nebulizer demonstrated the highest delivery efficiency when calculated as a percentage of the loading dose, whereas the PARI BOY SX + LC SPRINT (breath-enhanced) jet nebulizer had the highest delivery efficiency when calculated as a percentage of the emitted dose.

**Conclusion:**

The results suggest that the total inspiration volume, output rate, and particle size should be considered when IgG nebulization is used. We, therefore, propose a method for evaluating the efficiency of nebulizer for predicting antibody drug delivery.

## Introduction

Nebulizers are typically used to manage respiratory diseases, such as asthma, chronic obstructive pulmonary disease, cystic fibrosis, and pneumonia both in hospitals and homes [1–3]. These medical devices aerosolize drugs and deliver them rapidly via the respiratory tract. There are different types of nebulizers available, including jet, ultrasound, and mesh (static and vibrating) nebulizers, classified based on their operating principles [1, 2]. Jet nebulizers are reliable medical devices that nebulize drugs using compressor-supplied air. However, the need for a compressor means that jet nebulizers are heavy, noisy, and vibrate during use. Furthermore, although the output rates are high, deviations in the aerosol mass distribution are large [4–6]. Conversely, ultrasonic nebulizers produce aerosols using a piezoelectric crystal that vibrates at high frequencies; however, they have limitations such as large residual volumes, degradation of heat-sensitive materials, and inability to aerosolize viscous solutions [2]. Static mesh nebulizers incorporate a mesh in front of a horn attached to a vibrating piezoelectric element; horn vibrations are transmitted to drugs in contact with the mesh [7]. Vibrating mesh nebulizers possess a micro-sized perforated mesh, piezoelectric element, and ring actuator. Mesh vibrations cause drugs to flow into the mesh holes, producing aerosols on the opposite side.

The drug delivery efficiency of nebulizer-based aerosol therapies depends on patient and respiratory disease characteristics, breathing patterns, inspiration/expiration volumes, and airway diameter [8, 9]. Unlike oral or intravenous therapy, nebulizer therapy delivers drugs directly to the inner lumen of the airways and treatment site, reducing the systemic dose of most aerosolized drugs compared to oral administration and intravenous injections. This leads to fewer side effects [10, 11]. Therapeutic antibodies are one of the drugs used to treat asthma [12, 13]. Benralizumab and mepolizumab are newly developed therapies for severe eosinophilic asthma and are humanized immunoglobulin G (IgG) antibodies targeting the IL-5 receptor and IL-5, respectively, thereby suppressing the corresponding pathway [14]. Inhaled antibody therapy has the potential to enhance the vaccine-induced response to respiratory viruses by providing a rapid neutralization response. Because inhaled antibodies are delivered directly to the lungs and nose, they can be used to treat localized areas of the respiratory tract. Preclinical studies have reported that antibody nebulization therapy is more effective than other therapeutic routes [15–17].

IgG nebulization requires the consideration of several factors, such as heat sensitivity, nebulizer type, and drug viscosity [18]. Furthermore, nebulizer drug delivery can be conveniently and reproducibly predicted using in vitro simulations [19]. The hypothesis of this study is that the drug delivery efficiency will be different depending on the airway model, breathing pattern, and nebulizer types. In this study, a breathing simulator that met the volume control requirements of the ventilator standard was employed to evaluate the delivery efficiency of IgG nebulization using five nebulizers. Adult and child airway models were tested using normal and asthmatic breathing patterns.

## Materials and methods

### Nebulizers

A single unit of five nebulizers was tested in this study. The nebulizer types and abbreviations are listed in Table 1. The PARI BOY SX + LC SPRINT nebulizer was used with two nozzles (red or blue) supplied by the manufacturer. The size of aerosols generated by each nebulizer was determined based on data from a previous study. The aerosol size was measured by analyzing the distribution of IgG aerosol size (Mass Median Diameter, MMD) using the laser diffraction method with a Spraytec (Malvern instrument, Malvern, UK), and the volume median diameter (Dv50) was subsequently calculated. The measurement was performed under open ambient condition, with the nebulizer outlet positioned 5 cm away from the laser beam passing through the laser diffraction measurement zone. We utilized the particle size data obtained by aerosolizing 1 mg/mL of IgG with each nebulizer, which has been identified in the previous study. The particle sizes from nebulizers JN-PARIr, JN-PARIb, SMN-U150, VMN-SM1, and VMN-SM3 were 3.21, 5.55, 6.69, 4.72, and 6.75 μm, respectively [20].

**Table 1:** Tested nebulizers

Type	Models	Abbreviation
Jet	PARI BOY SX + LC SPRINT (breath-enhanced) red nozzle (PARI GmbH, Starnberg, Germany)	JN-PARIr
	PARI BOY SX + LC SPRINT (breath-enhanced) blue nozzle (PARI GmbH, Starnberg, Germany)	JN-PARIb
Static mesh	NE-U150 (Omron Healthcare, Kyoto, Japan)	SMN-U150
Vibrating mesh	NE-SM1 NEPLUS (KTMED Co, Seoul, Korea)	VMN-SM1
	NE-SM3 (KTMED Co, Seoul, Korea)	VMN-SM3

### Breathing simulator

The breathing simulator used in this study was developed in our laboratory, as previously described [21]. It comprised a linear actuator, stepper motor, motor driver, air cylinder, airway model, and disposable filter. The speed and movement distance of the linear actuator were controlled using an Arduino Uno (Arduino.cc, Ivrea, Italy), allowing for the generation of desired breathing patterns. The air cylinder was designed to mimic the function of the human lung and had a capacity compatible with human breathing volumes. A disposable filter (Pro-guard EX; GMS Korea, Bucheon, Korea) was used to collect the drugs entering the air cylinder in the simulator.

### Breathing patterns

The drug delivery efficiency was tested using five breathing patterns: ISO 27427:2013 [22], normal adult [9], asthmatic adult [9], normal child [23], and asthmatic child [24]. The inspiration: expiration (I:E) ratio, respiration rate, tidal volume, and inspiration volume for each pattern are listed in Table 2. For the asthmatic child, the I:E ratio was not determined based on spontaneous breathing but rather followed was generally proposed mechanical ventilator setting.

**Table 2:** Breathing patterns generated by the breathing simulator

Breathing pattern	Inspiration: Expiration (I:E) ratio	Respiration rate (BPM)	Tidal volume (mL)	Inspiration volume (mL/min)
ISO 27427	1:1	15	500	3,750
Normal adult	1:2	15	500	2,500
Asthma adult	1:2.5	25	290	2,071
Normal child	1:2	20	200	1,333
Asthma child	1:4	23	200	920

The average volume error of the breathing patterns was 1.46 ± 0.73 %

### Airway models

A VTA-M (RDDonline, Richmond, USA) adult airway model was used in this experiment. This realistic throat model was developed based on adult Computed Tomography data and validated by Virginia Commonwealth University [25, 26] and is suitable for clinically relevant in vitro testing. The child airway model used in this study was developed by the Pharmaceutical Physics Laboratory of Boehringer Ingelheim Pharma GmbH & Co. KG [27, 28]. A ProJet 6000 (3D Systems, South Carolina, USA) was used to print the realistic 3D model of five-year-old pediatric drawings released by RDDonline as open source. The model was constructed using Accura ClearVue (3D Systems, Rock Hill, South Carolina, USA), which is the same material used for the adult airway model.

### Drug

Human IgG (100 mg/mL) was purchased from GC Biopharma Corp. (Yongin, Korea) and prepared at concentrations of 1, 10, 20, or 40 mg/mL with saline. The viscosities at IgG concentrations of 1, 10, 20, and 40 mg/mL were approximately 0.97, 1.03, 1.12, and 1.33 mPa-s, respectively. A m-VROC™ viscometer (RheoSense Inc., CA, USA) was operated at 25°C to measure the viscosity of each IgG solution.

### Nebulization performance and drug delivery efficiency evaluation

The nebulizers were loaded with 2 mL of IgG solution at concentrations of 1, 10, 20, and 40 mg/mL, and the residual volumes, nebulization times, and output rates were measured. The jet nebulizers were operated for 1 min after sputtering, whereas the static and vibrating-mesh nebulizers were operated until no aerosol was visible. The residual volumes were obtained by measuring the weight of nebulizer before and after nebulization using an analytical balance. Output rates (mL/min) were calculated using the following formula [21]:

$$\mathrm{Output \; Rate }\ (\mathrm{mL}/\mathrm{min})=\frac{\mathrm{Loading \; Volume }\ (\mathrm{mL}) -\mathrm{ Residual \; Volume }\ (\mathrm{mL})}{\mathrm{Nebulization \; Time }\ (\mathrm{min})}$$

### Delivered drug collection

A delivered drug has been collected from the drug deposited in the filter, any drug deposited in the mouth-throat was not considered. To collect the IgG from filter, filter was sealed in a zip-lock bag containing 20 mL of saline with 0.5 % SDS. The bag was then heated for 2 min at 60 °C and shaken for 2 h to elute the IgG [29]. The eluted IgG solution (1 mg/mL) was quantified using a Micro BCATM Protein Assay Kit (Thermo Scientific, MA, USA). IgG concentrations of 10, 20, and 40 mg/mL were quantified at 280 nm using quartz cuvettes and a microplate reader (SpectraMax Plus 384, Molecular Devices, Sunnyvale, CA, USA). This process was repeated three times for the nebulizers.

### Drug delivery efficiency evaluation

Drug delivery efficiency was calculated by expressing the delivered dose (DD, mg) as a percentage of the loading dose (LD, mg) and emitted dose (ED, mg), expressed as DD/LD (%) and DD/ED (%), respectively. However, it is important to note that in this experiment, DD represents post-throat filter dose and does not reflect the amount of drug that penetrates the extrathoracic region, rather it represents the amount that reaches the patient. For LD, 2 mL of each IgG concentration (1, 10, 20, and 40 mg/mL) were used. DD is a measure of the total amount of IgG collected using the disposable filter. ED is the value obtained by subtracting the residual volume from the loaded volume (ED = loaded volume – residual volume). Some types of nebulizers are known to be associated with an increase in solute concentration in the reservoir as nebulization time due to solvent evaporation. Therefore, this effect may introduce a bias in the estimation of the ED [30]. DD/LD is a general parameter indicating the ratio of the amount of drug collected in the filter to the amount of drug used, whereas DD/ED considers the nebulizer residual volume and reflects the ratio of drugs collected in the filter to the ED; they are calculated using the following formulae [21]:

$$\begin{array}{c}\mathrm{DD}/\mathrm{LD }\left(\mathrm{\%}\right)= \frac{\mathrm{Delivered \; Dose\ } \left(\mathrm{mg}\right)}{\mathrm{Loading \; Dose\ } \left(\mathrm{mg}\right)}\times 100 \\ \mathrm{DD}/\mathrm{ED}\ (\mathrm{\%}) = \frac{\mathrm{Delivered \; Dose\ } \left(\mathrm{mg}\right)}{\mathrm{Emitted\; Dose\ } \left(\mathrm{mg}\right)}\times 100\end{array}$$

The residual volume cannot be nebulized also results in wastage of drug. The difference between DD/LD (%) and DD/ED (%) reflects the residual volume the nebulizer, the larger the difference, the greater the residual volume. The residual volume is mainly caused by the operating principle or structure of the nebulizer, so it is irrelevant to the particle size and output rate that affect nebulization performance. DD/ED was suggested to express the drug delivery efficiency based on the emitted drug as it was affected by nebulizing performance of the device, such as particle size and output rate.

### Statistical analysis

Statistical analysis was performed using a one-way analysis of variance (ANOVA) followed by Dunnett's post hoc test in SigmaPlot Ver. 12.5 (Systat Software Inc, Chicago, USA). Results are presented as means ± SDs; statistical significance was accepted for *p* values < 0.05.

## Results

### Nebulization performance of IgG in various nebulizers

Table 3 shows that IgG nebulization performance of the five nebulizers was evaluated by measuring the residual volume, nebulization time, and output rate at concentrations of 1, 10, 20, and 40 mg/mL, applying the breathing patterns given in ISO 27427:2013. There were no changes in residual volume except for40 mg/ml of VMN-SM1. Nebulization time increased for all devices at 40 mg/ml and with JN-PARIr, VMN-SM1, and VMN-SM3 at 20 mg/ml. Output rate decreased for most devices at 40 mg/ml concentrations except for jet nebulizers.

**Table 3:** Nebulization performance of the five nebulizers with varying IgG concentrations in ISO breathing pattern

IgG conc. (mg/mL)	Nebulizer	Residual volume (%)	Nebulization time (min)	Output rate (mL/min)
1	JN-PARIr	37.7 ± 1.1	5.6 ± 0.1	0.225 ± 0.00
	JN-PARIb	30.9 ± 0.6	4.4 ± 0.1	0.315 ± 0.01
	SMN-U150	14.1 ± 2.8	5.2 ± 0.7	0.334 ± 0.02
	VMN-SM1	9.5 ± 2.3	6.7 ± 0.1	0.269 ± 0.01
	VMN-SM3	8.7± 2.0	5.2 ± 0.1	0.351 ± 0.01
10	JN-PARIr	35.4 ± 4.1	5.8 ± 0.8	0.223 ± 0.02
	JN-PARIb	35.6 ± 7.0	4.6 ± 0.2	0.282 ± 0.05
	SMN-U150	15.7 ± 0.7	5.3 ± 0.2	0.317 ± 0.01
	VMN-SM1	13.6 ± 3.0	7.1 ± 0.6	0.246 ± 0.02
	VMN-SM3	15.1 ± 2.4	5.4 ± 0.1	0.314 ± 0.01*
20	JN-PARIr	34.6 ± 3.0	6.1 ± 0.9*	0.216 ± 0.02
	JN-PARIb	32.2 ± 1.9	4.9 ± 0.8	0.275 ± 0.02
	SMN-U150	16.2 ± 2.4	5.8 ± 0.8	0.290 ± 0.01
	VMN-SM1	11.6 ± 1.7	7.4 ± 0.2*	0.239 ± 0.00*
	VMN-SM3	13.1 ± 0.7	6.3 ± 0.1*	0.275 ± 0.01*
40	JN-PARIr	28.2 ± 4.0	7.2 ± 0.9*	0.202 ± 0.02
	JN-PARIb	24.6 ± 1.8	5.9 ± 0.7*	0.258 ± 0.02
	SMN-U150	10.4 ± 4.4	6.4 ± 0.7*	0.281 ± 0.03*
	VMN-SM1	19.6 ± 1.6*	8.1 ± 0.9*	0.199 ± 0.01*
	VMN-SM3	13.7 ± 5.8	7.6 ± 0.7*	0.229 ± 0.01*

All data are presented as the mean ± SD values. * *p* < 0.05 versus 1 mg/mL of IgG

### Delivery efficiency of IgG in various nebulizers: adult airway model

Table 4 presents the delivered dose (DD) of IgG by the different nebulizers at various concentrations in adult breathing patterns. The DD of IgG by the five nebulizers in the adult asthma breathing pattern decreased by amounts ranging from 58.9 % to 85.7 % when compared to that in normal adult breathing pattern at all IgG concentrations. In ISO 27427, the DD values of all five nebulizers ranged between 93.1 % and 155.5 % when compared to normal adult breathing pattern at all IgG concentrations; they were mostly higher than those of normal adult breathing pattern. Table 5 displays the DD/LD values of the adult breathing patterns. The DD/LD for the adult asthma breathing pattern decreased by amounts ranging from 58.6 % to 91.2 % when compared to that for the normal adult breathing pattern at all IgG concentrations. Table 6 shows the DD/ED values for adult breathing patterns, indicating that the DD/ED for the adult asthma breathing pattern decreased by amounts ranging from 59.3 % to 85.0 % when compared to that for the normal adult breathing pattern at all IgG concentrations. In all breathing patterns, VMN-SM1 showed the highest DD/LD when calculated as LD without considering residual volume. On the other hand, since JN-PARIr has a large residual volume, the DD/ED value was the highest when calculated as ED considering the residual volume.

**Table 4:** Delivered dose (DD, mg) for adult breathing patterns. The DD values of the five nebulizers for the adult asthma breathing pattern decreased compared to those for the normal adult breathing pattern at all IgG concentrations

**Breathing pattern**	**IgG conc. (mg/mL)**	**JN-PARIr (mg)**	**JN-PARIb (mg)**	**SMN-U150 (mg)**	**VMN-SM1 (mg)**	**VMN-SM3 (mg)**
ISO 27427	1	0.506 ± 0.04	0.489 ± 0.07	0.436 ± 0.02*	0.539 ± 0.04	0.278 ± 0.02
	10	5.250 ± 0.16*	4.795 ± 0.13*	4.386 ± 0.10*	5.433 ± 0.44	2.814 ± 0.12*
	20	10.843 ± 0.64*	10.421 ± 0.50*	9.504 ± 0.36*	11.830 ± 0.14*	7.426 ± 0.06*
	40	18.049 ± 1.73	19.659 ± 1.57	19.137 ± 1.14	20.248 ± 1.43	12.435 ± 0.18
Normal adult	1	0.498 ± 0.02	0.477 ± 0.02	0.374 ± 0.01	0.579 ± 0.01	0.298 ± 0.03
	10	4.831 ± 0.10	4.036 ± 0.08	3.224 ± 0.15	5.277 ± 0.20	2.538 ± 0.13
	20	8.021 ± 0.09	7.234 ± 0.24	6.684 ± 0.23	8.714 ± 0.11	5.647 ± 0.18
	40	17.281 ± 0.39	12.639 ± 0.28	13.972 ± 0.50	18.136 ± 0.81	10.651 ± 0.35
Asthma adult	1	0.355 ± 0.02*	0.336 ± 0.02*	0.305 ± 0.02*	0.472 ± 0.02*	0.239 ± 0.01*
	10	2.889 ± 0.04*	2.645 ± 0.18*	2.404 ± 0.11*	3.107 ± 0.05*	1.797 ± 0.08*
	20	6.443 ± 0.51*	6.048 ± 0.46*	4.797 ± 0.18*	7.467 ± 0.11*	4.392 ± 0.13*
	40	11.910 ± 0.41*	10.349 ± 0.65*	9.318 ± 0.26*	12.566 ± 0.22*	6.821 ± 0.57*

All data are presented as the mean ± SD values. * *p* < 0.05 versus the normal adult pattern

**Table 5:** Delivered dose (DD, mg)/Loading dose (LD, mg) for adult breathing patterns. The DD/LD (%) values of the five nebulizers for the asthma adult breathing pattern decreased compared to those for the normal adult breathing pattern at all IgG concentrations

**Breathing pattern**	**IgG conc. (mg/mL)**	**JN-PARIr (%)**	**JN-PARIb (%)**	**SMN-U150 (%)**	**VMN-SM1 (%)**	**VMN-SM3 (%)**
ISO 27427	1	25.21 ± 1.75	24.31 ± 3.23	21.69 ± 1.05*	26.90 ± 1.82	0.278 ± 0.80
	10	26.11 ± 0.78*	23.89 ± 0.64*	21.92 ± 0.50*	27.11 ± 2.28	2.814 ± 0.63*
	20	26.96 ± 1.52*	25.99 ± 1.28*	23.64 ± 0.99*	29.47 ± 0.45*	7.426 ± 0.14*
	40	22.40 ± 2.19	24.33 ± 1.98*	23.85 ± 1.44*	25.25 ± 1.85	12.435 ± 0.24*
Normal adult	1	24.83 ± 1.13	23.77 ± 1.00	18.61 ± 0.57	28.80 ± 0.34	0.298 ± 1.25
	10	24.12 ± 0.47	20.13 ± 0.39	16.03 ± 0.73	26.30 ± 1.04	2.538 ± 0.65
	20	19.97 ± 0.26	17.99 ± 0.67	16.65 ± 0.60	21.71 ± 0.28	5.647 ± 0.45
	40	21.53 ± 0.42	15.73 ± 0.34	17.40 ± 0.68	22.61 ± 1.00	10.651 ± 0.44
Asthma adult	1	17.71 ± 1.10*	16.74 ± 0.86*	15.21 ± 1.28*	23.53 ± 1.08*	0.239 ± 0.52*
	10	14.41 ± 0.20*	13.19 ± 0.93*	12.00 ± 0.57*	15.49 ± 0.26*	1.797 ± 0.40*
	20	16.02 ± 1.31*	15.02 ± 1.14*	11.94 ± 0.44*	18.58 ± 0.30*	4.392 ± 0.32*
	40	14.78 ± 0.54*	12.82 ± 0.73*	11.61 ± 0.32*	15.69 ± 0.27*	6.821 ± 0.72*

All data are presented as the mean ± SD values. * *p* < 0.05, versus the normal adult pattern

**Table 6:** Delivered dose (DD, mg)/Emitted dose (ED, mg) for adult breathing patterns. The DD/ED (%) values of the five nebulizers for the asthma adult breathing pattern decreased compared to those for the normal adult breathing pattern at all IgG concentrations

**Breathing pattern**	**IgG conc. (mg/mL)**	**JN-PARIr (%)**	**JN-PARIb (%)**	**SMN-U150 (%)**	**VMN-SM1 (%)**	**VMN-SM3 (%)**
ISO 27427	1	40.48 ± 3.11*	35.15 ± 4.38	25.27 ± 1.12*	29.72 ± 1.78	15.17 ± 0.55
	10	40.51 ± 1.74*	37.33 ± 3.22*	26.00 ± 0.60*	31.35 ± 1.63	16.55 ± 0.66*
	20	41.23 ± 0.44*	38.32 ± 1.15*	28.23 ± 1.61*	33.33 ± 0.50*	21.30 ± 0.04*
	40	31.14 ± 1.53*	32.32 ± 3.22*	26.62 ± 1.26*	31.46 ± 2.81	18.02 ± 1.43*
Normal adult	1	34.50 ± 2.10	34.44 ± 1.56	21.71 ± 0.79	31.54 ± 1.09	17.17 ± 3.35
	10	34.43 ± 0.76	29.24 ± 0.78	19.72 ± 0.95	29.13 ± 0.89	14.29 ± 0.73
	20	29.13 ± 0.95	26.78 ± 0.97	20.03 ± 0.81	24.82 ± 0.28	16.34 ± 0.44
	40	35.37 ± 1.63	23.91 ± 0.40	20.48 ± 1.14	28.03 ± 0.28	15.56 ± 0.57
Asthma adult	1	26.91 ± 2.27*	25.63 ± 0.30	17.76 ± 2.19*	26.16 ± 1.35*	13.42 ± 0.40
	10	21.70 ± 0.32*	20.66 ± 0.80*	14.91 ± 0.89*	17.28 ± 0.27*	10.26 ± 0.36*
	20	23.36 ± 2.32*	22.71 ± 1.78*	14.83 ± 0.33*	21.09± 0.37*	12.44 ± 0.28*
	40	22.59 ± 1.29*	19.26 ± 0.85*	15.40 ± 0.63*	19.20 ± 0.37*	10.51 ± 0.21*

All data are presented as mean ± SD values. * *p* < 0.05, versus the normal adult pattern

### Delivery efficiency of IgG in various nebulizers: child airway model

Table 7 presents the DD of IgG through various nebulizers in the child breathing patterns at different concentrations of IgG. The DD of IgG by the five nebulizers in the child asthma breathing pattern decreased by amounts ranging from 58.6 % to 91.2 % when compared to those in the normal child at all IgG concentrations. Table 8 presents the DD/LD values of the child breathing patterns, which decreased by amounts ranging from 58.6 % to 91.2 % in the child asthma breathing pattern when compared to those in the normal child breathing pattern at all IgG concentrations. Similarly, Table 9 shows the DD/ED values for the child breathing patterns, which decreased by amounts ranging from 59.0 % to 92.6 % at all IgG concentrations for the child asthma breathing pattern when compared to those for the normal child breathing pattern. Similar to the adult breathing patterns, VMN-SM1 had the highest DD/LD, whereas JN-PARIr had the highest DD/ED.

**Table 7:** Delivered dose (DD, mg) for child breathing patterns. The DD values of the five nebulizers for the child asthma breathing pattern decreased compared to those for the normal child breathing pattern at all IgG concentrations

**Breathing pattern**	**IgG conc. (mg/mL)**	**JN-PARIr (mg)**	**JN-PARIb (mg)**	**SMN-U150 (mg)**	**VMN-SM1 (mg)**	**VMN-SM3 (mg)**
Normal child	1	0.280 ± 0.01	0.197 ± 0.02	0.256 ± 0.00	0.422 ± 0.00	0.253 ± 0.01
	10	2.353 ± 0.13	1.591 ± 0.08	1.937 ± 0.11	2.695 ± 0.21	1.700 ± 0.14
	20	5.199 ± 0.37	4.260 ± 0.44	4.724 ± 0.40	5.856 ± 0.09	3.648 ± 0.13
	40	8.787 ± 0.21	6.101 ± 0.54	7.194 ± 0.33	11.013 ± 0.78	5.864 ± 0.69
Asthma child	1	0.212 ± 0.01*	0.135 ± 0.00*	0.190 ± 0.02*	0.297 ± 0.01*	0.216 ± 0.01*
	10	2.017 ± 0.07*	1.419 ± 0.14	1.572 ± 0.06*	2.457 ± 0.20	1.256 ± 0.16*
	20	3.584 ± 0.10*	2.495 ± 0.18*	3.045 ± 0.14*	4.105 ± 0.13*	2.352 ± 0.08*
	40	7.118 ± 0.32*	5.234 ± 0.15	6.012 ± 0.08*	7.883 ± 0.11*	4.939 ± 0.24

All data are presented as the mean ± SD values. * *p* < 0.05, versus the normal child pattern

**Table 8:** DD/LD (%) for child breathing patterns. The DD/LD (%) values of the five nebulizers for the child asthma breathing pattern decreased compared to those for the normal child breathing pattern at all IgG concentrations

**Breathing pattern**	**IgG conc. (mg/mL)**	**JN-PARIr (%)**	**JN-PARIb (%)**	**SMN-U150 (%)**	**VMN-SM1 (%)**	**VMN-SM3 (%)**
Normal child	1	13.93 ± 0.40	9.80 ± 1.11	12.77 ± 0.19	21.11 ± 0.17	0.253 ± 0.55
	10	11.71 ± 0.71	7.91 ± 0.39	9.66 ± 0.51	13.44 ± 1.04	1.700 ± 0.69
	20	12.93 ± 0.93	10.60 ± 1.09	11.77 ± 1.00	14.59 ± 0.22	3.648 ± 0.32
	40	10.95 ± 0.26	7.61 ± 0.69	8.96 ± 0.41	13.72 ± 0.93	5.864 ± 0.85
Asthma child	1	10.48 ± 0.43*	6.73 ± 0.18	9.46 ± 0.78*	14.75 ± 0.52*	0.216 ± 0.42*
	10	10.04 ± 0.30*	7.06 ± 0.66	7.84 ± 0.29*	12.26 ± 0.98	1.256 ± 0.78*
	20	8.93 ± 0.27	6.22 ± 0.43*	7.58 ± 0.34*	10.23 ± 0.34*	2.352 ± 0.20*
	40	8.84 ± 0.41*	6.53 ± 0.19	7.50 ± 0.09	9.85 ± 0.14*	4.939 ± 0.30

All data are presented as the mean ± SD values. * *p* < 0.05, versus the normal child pattern

**Table 9:** DD/ED (%) for the child breathing patterns. The DD/ED (%) values of the five nebulizers for the child asthma breathing pattern decreased compared to those for the normal child breathing pattern at all IgG concentrations

**Breathing pattern**	**IgG conc. (mg/mL)**	**JN-PARIr (%)**	**JN-PARIb (%)**	**SMN-U150 (%)**	**VMN-SM1 (%)**	**VMN-SM3 (%)**
Normal child	1	23.20 ± 1.00	16.53± 1.29	15.29 ± 0.50	23.09 ± 0.38	13.85 ± 0.62
	10	18.21 ± 0.32	12.54 ± 0.55	11.08 ± 0.41	14.90 ± 0.83	9.55 ± 0.83
	20	20.40 ± 0.61	10.65 ± 1.84	14.53 ± 1.71	16.57 ± 0.51	10.18 ± 0.46
	40	17.69 ± 1.42	11.61 ± 1.73	11.94 ± 0.76	16.87 ± 0.80	9.22 ± 1.01
Asthma child	1	17.51 ± 1.24*	10.27 ± 0.32*	11.57 ± 1.74*	16.41 ± 0.62*	12.78 ± 0.79
	10	15.74 ± 0.42	11.61 ± 1.65	9.45 ± 0.67*	13.70 ± 1.07	7.15 ± 0.89
	20	14.92 ± 0.87*	10.27 ± 0.36*	8.99 ± 0.16*	11.61 ± 0.20*	6.64 ± 0.22*
	40	14.91 ± 0.58*	10.81 ± 0.83	9.77 ± 0.44*	12.18 ± 0.07*	7.83 ± 0.35

All data are presented as the mean ± SD values. * *p* < 0.05, versus the normal child pattern

### Comparison of DD, DD/LD, and DD/ED between adult and child

The DD values of all five nebulizers in the normal child breathing pattern ranged from 39.4 % to 84.9 % compared to those in the normal adult breathing pattern at all IgG concentrations. Similarly, the DD values in the child asthma breathing pattern from 40.2 % to 90.3 % when compared to those of the adult asthma at all IgG concentrations. The DD/LD values of all five nebulizers in the normal child breathing pattern ranged from 39.3 % to 85.2 % when compared with those in the normal adult breathing pattern at all IgG concentrations. Similarly, the DD/LD values in the child asthma breathing pattern ranged from 40.2 % to 90.3 % of those in the adult asthma values at all IgG concentrations. The DD/ED values of all five nebulizers in the normal child breathing pattern ranged from 42.9 % to 80.7 % when compared to those in the normal adult breathing pattern at all IgG concentrations. In the child asthma breathing pattern, DD/EDs were measured from 41.6 % to 95.2 % when compared to those in the adult asthma breathing pattern at all IgG concentrations. The differences in DD, DD/LD, and DD/ED between the adult and child breathing pattern mainly depended on the inspiration volume.

### Relationship between DD and total inspiration volume, output rate, and particle size

To investigate the factors influencing DDs, the relationships among inspiration volume, output rate, and particle size were evaluated at an IgG concentration of 1 mg/mL. The total inspiratory volume was calculated by multiplying the inspiratory volume (mL/min) by the nebulization time, whereas the inspiratory volume was calculated by multiplying the tidal volume by the inspiratory ratio of the respiratory cycle and respiration rate (BPM).

$$\mathrm{Total inspiration Volume }\left(\mathrm{mL}\right)=\mathrm{Inspiration Volume }\left(\mathrm{mL}/\mathrm{min}\right)\times \mathrm{ Nebulization time }(\mathrm{min})$$

**Fig. 1:**
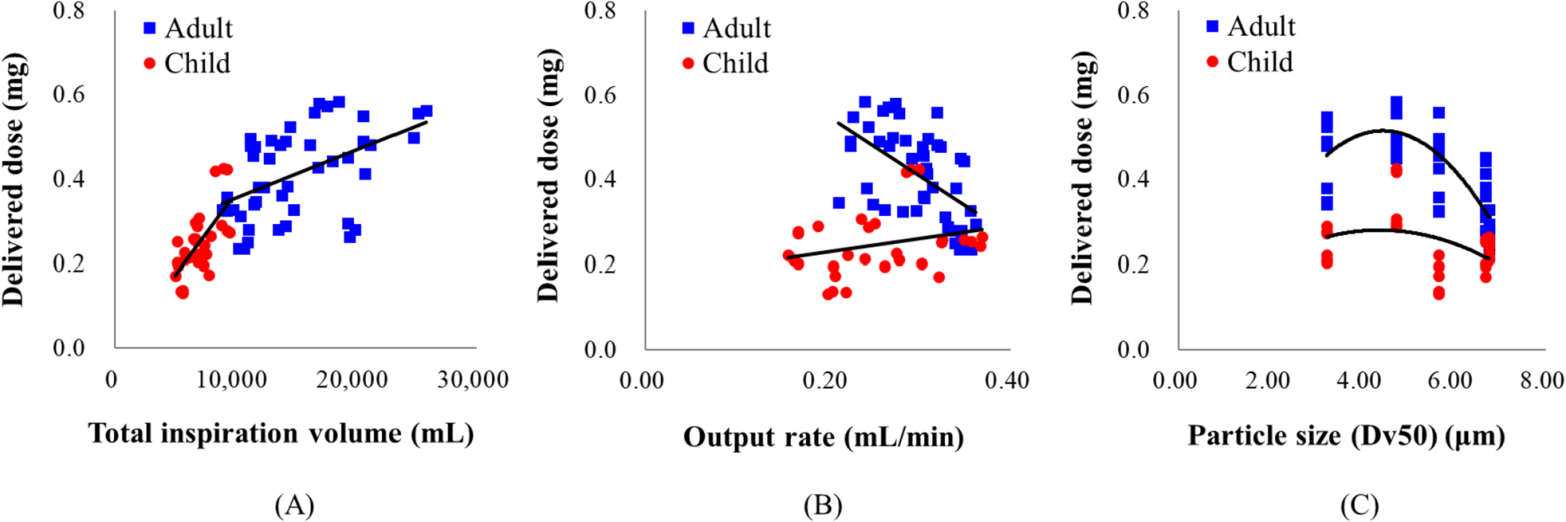
Relationships between the delivered dose (DD) and total inspiration volume (**A**), output rate (**B**), and particle size (**C**)

The total inspiration volumes for each breathing pattern decreased in the order: ISO 27427, normal adult, asthma adult, normal child, and asthma child breathing pattern. Figure 1 shows the relationship between the DD and total inspiration volume, output rate, and particle size. In the adult and child breathing pattern, the correlation coefficients of total inspiration volume and DD were 0.49 and 0.68, respectively; the DD increased as the total inspiration volume increased. The correlation coefficient between the output rate and DD in the adult was -0.56, and as the output rate increased, the DD decreased. This correlation was less prominent in the child with a coefficient of 0.26. The correlation coefficients between particle size and DD in the adult and child were -0.58 and -0.27, respectively; the DD decreased when the particle size exceeded 5 μm. The differences in the IgG-delivered dose between adults and child seem to be due to the differences in total inspiration, as shown in Fig. 1. More IgG was inhaled at larger inspiratory volumes, resulting in higher doses being delivered.

## Discussion

A comparison of the drug delivery efficiencies of different nebulizer types is difficult because the output rates, particle sizes, and residual volumes vary considerably. The DD/LD ratio provides a measure of delivery efficiency based on the total amount of drug used, whereas the DD/ED ratio considers the residual volume of the nebulizer. We, therefore, evaluated the drug delivery efficiency of the five nebulizers using the DD/LD and DD/ED. Jet nebulizers had higher DD/ED values than other nebulizers across all breathing patterns and concentrations, but larger residual volumes. In contrast, mesh nebulizers had lower residual volumes and higher DD/LD values than jet nebulizers. Although VMN-SM3 and SMN-U150 had higher output rates than VMN-SM1, they exhibited lower DD than VMN-SM1. This implies that higher output rates do not guarantee higher delivered doses.

IgG is incompletely nebulized in mesh nebulizers because of the foam generated by acoustic cavitation, which is caused by a drop in local pressure below the vapor pressure [31]. It was previously shown that mesh nebulizer IgG output rates are negatively related to viscosity, which is consistent with our observation that the output rate decreased with concentration. Moreover, nebulization performance depends on drug molecular properties [29].

Vonarburg et al. showed that 2-fold higher IgG concentration of loading dose increase almost double of the delivered dose, when nebulized IgG with electronic vibrating membrane nebulizer (eFlow®, PARI Pharma GmbH, Germany) in an ISO breathing pattern [29]. This result is consistent with those of the jet and mesh nebulizers used for IgG nebulization in this study. They also reported that the output rate decreased and the nebulization time increased as the concentration of IgG increased, this phenomenon consistent in the mesh nebulizers used in our study [29]. The delivered dose (%) result from Vonarburg was higher than our delivered dose. Because their breathing simulation experiment connected the filter directly between the mouthpiece and pump without an airway model, IgG was captured more. Also, the mesh nebulizer used in the Vonarburg study was designed to generate a high output rates as well as a big mixing chamber (not yet commercially available). Particles with an aerodynamic diameters greater than 5 μm generally tend to deposit through impaction in the mouth and upper airways, whereas particles within the 2 to 5 μm range are most effective at reaching the deeper lung regions [22]. It appears that the deposition in the mouth and throat regions contributes to a decrease in the delivered dose for larger particle sizes (Fig. 1).

The delivery efficiency of Ventolin has been previously investigated using adult breathing patterns and an airway model [21]. A similar drug delivery tendency was observed in this study, which was related to the I:E ratio, tidal volume, and respiration rate. The doses of IgG delivered in this study were slightly higher than those reported for Ventolin; however, quantitative comparisons were not possible because different airway models were used. The airway model used in previous studies was based on the hydraulic diameters measured from a replica human oral airway cast of a healthy male adult. The computational domain addressed the oral cavity, soft palate, pharynx, larynx, and trachea [32]. In contrast, the airway model used in the present study added the structure of the triangular glottis and throat entrance, and the differences between the airway model structures and dimensions affected aerosol deposition in the airway pathway and flow resistance. Furthermore, the surface adsorption characteristics differed because different materials were used to create the airway models.

It has been previously reported that slow, deep breathing can increase drug delivery to the lungs [33]. We used the ISO 27427 deep, which had the lowest respiration rate and the longest inspiration phase. We observed that the increase in total inspiration volume increased DD, and the DD/LD for ISO 27427 was higher than that for the other breathing patterns. However, we also found that increasing the output rate did not necessarily increase the DD. Drugs that do not enter the airway path during the expiration phase are wasted; therefore, it is important to consider a suitable nebulizer output rate, inspiration rate, and total inspiration volume based on the specific drug composition to achieve effective medication for patients while minimizing drug waste.

Nevertheless, this study has several limitations that should be acknowledged. First, the breathing of patients with asthma can be irregular in terms of breathing cycles and tidal volume in clinical settings. However, the breathing simulator in this study consistently generated accurate tidal volumes and breathing cycles. Second, our model did not take into account the variability in structures of the patients' airways due to the disease type and age, since a single unit of each nebulizer was used, the findings may not be representative of the general population or applicable to other devices. Fourth, the drugs remained in the airway model was not evaluated in this study. In future studies, bronchial trees, realistic breathing patterns, mouthpieces, masks, and airway structures of patients with different diseases should be considered to simulate clinical situations more closely.

## Conclusion

In this study, the drug delivery efficiencies of five nebulizers were compared using five breathing patterns, four IgG concentrations, and two airway models. The results confirmed that the delivered dose and drug delivery efficiency were lower in the child compared to those in the adult and in asthma than in the normal breathing pattern. This evaluation method suggests various breathing patterns with different I:E ratios, respiration rates, tidal volumes, and inspiration volumes to assess the efficiency of nebulizer in delivering IgG. The delivered was proportional to the total inhalation volume but not to the output rate and particle size. Based on these results, we propose a method for evaluating the drug delivery efficiency of nebulizing antibody drugs that can be utilized to determine the expected dose in clinical settings.

## Data Availability

Not applicable.
